# Minding the gap: a sex difference in young infants’ mental rotation through thirty degrees of arc

**DOI:** 10.3389/fpsyg.2024.1415651

**Published:** 2024-09-13

**Authors:** David S. Moore, Dawn Michele Moore, Scott P. Johnson

**Affiliations:** ^1^Psychology Field Group, Pitzer College, Claremont, CA, United States; ^2^Center for Advanced Study in the Behavioral Sciences, Stanford University, Stanford, CA, United States; ^3^Department of Psychology, New Mexico State University, Las Cruces, NM, United States; ^4^Department of Psychology, University of California, Los Angeles, Los Angeles, CA, United States

**Keywords:** mental rotation, spatial cognition, infant development, sex differences, infant cognitive development

## Abstract

Mental rotation (MR) is an important feature of spatial cognition invoking mental imagery of an object’s appearance when viewed from a new orientation. Prior studies have revealed evidence of MR in infants, including a sex difference similar to that detected in older populations. Some of these studies used visual habituation methods whereby infants were familiarized with an object rotating through a 240° angle, followed by test trials showing either the habituation object or a mirror image object rotating through the previously unseen 120° angle. Significantly longer looking at either of these objects was taken to reflect infants’ ability to *recognize* the habituation object even when seen from a novel viewpoint, suggesting the capacity for MR. However, these infants’ responses could, in theory, be explained with reference to perceptual discrimination rather than MR, because the views of the habituation and test objects were very similar in some video frames. In the current study, we observed a diverse population of 5-month-olds (24 females, 24 males) for evidence of MR through 30° of arc. In this more challenging test, our stimuli left a 30° gap angle between critical video frames representing the habituation and test objects. Consistent with earlier reports, we found that relative to female infants, male infants looked significantly longer at the mirror image test stimulus immediately following habituation. These results add to an emerging consensus that some young infants are capable of MR, and that male and female infants on average behave differently in this type of MR task.

## Introduction

Mental rotation (MR) is an aspect of spatial cognition that involves imagining how an object would look if it were rotated in space into a new orientation. This is a useful ability with applications in domains as diverse as reading (Rusiak et al., 2007; Rüsseler et al., 2005), surgery (Conrad et al., 2006), navigation (Kerkman et al., 2000; Levine et al., 1999), architecture, dentistry, engineering, chemistry, cartography (Shea et al., 2001; Wai et al., 2009), and various branches of biology and mathematics (Frick, 2019; Lauer and Lourenco, 2016; Newcombe et al., 2019; van Tetering et al., 2019; Verdine et al., 2017; Young et al., 2018). Importantly, this kind of spatial skill early in life strongly predicts subsequent mathematical reasoning (Casey et al., 2015; Geer et al., 2019).

MR has been a subject of research since the early 1970s, when Shepard published now-classic results of chronometric studies that showed a direct relationship between the amount of *time* required to mentally rotate an object and the *angle* through which the object was to be mentally rotated (Shepard, 1978; Shepard and Metzler, 1971). Thus, after seeing an object from a particular perspective, an adult can recognize that object again faster after it has been rotated through a small angle than after it has been rotated through a large angle. These findings were considered consequential, as they were consistent with the claim that human cognition sometimes entails the use of analog representations of objects.

Subsequent studies revealed that, on average, men accurately complete MR tasks faster than women do (Linn and Petersen, 1985; Schöning et al., 2007). In fact, these tasks are associated with larger sex differences than any other spatial cognition tasks (Voyer et al., 1995). Effect sizes documenting sex differences in tasks requiring MR of 3-dimensional (3D) objects in 3D space are usually larger than effect sizes documenting sex differences in studies of aggressive behavior or rough-and-tumble play in childhood (Collaer and Hines, 1995), which are typically quite large. In the domain of cognition, these are the largest and most robust sex differences yet discovered (Linn and Petersen, 1985; Voyer et al., 1995).

### The development of MR competence

Studies on the development of MR early in life have repeatedly detected MR in populations between the ages of 5 and 12 years (e.g., Iachini et al., 2019; Kail, 1991; Kail et al., 1980; Marmor, 1975; Titze et al., 2010). However, some early studies of preschoolers did not find evidence of MR (Krüger et al., 2014; see, for example, Estes, 1998), suggesting that children less than 5 years of age lack this ability (Frick et al., 2013a; Quaiser-Pohl et al., 2010). Nonetheless, when simpler, developmentally appropriate tests of MR are used, it now appears that even some 3-and 4-year-old children can provide evidence of MR (Frick et al., 2013b; Krüger, 2018; Levine et al., 1999).

The study of MR in infancy began in earnest about 15 years ago. Unlike older participants, babies cannot respond appropriately to verbal instructions, so researchers have developed innovative techniques that permit inferences about this cognitive competence in pre-verbal populations. These techniques have built on older methods, including habituation or familiarization (e.g., Moore and Johnson, 2008; Quinn and Liben, 2008; Schwarzer et al., 2013a), change detection (e.g., Beckner et al., 2023b; Lauer et al., 2015), violation-of-expectation (e.g., Frick and Möhring, 2013; Hespos and Rochat, 1997; Möhring and Frick, 2013; Rochat and Hespos, 1996) and eye-tracking procedures (e.g., Pedrett et al., 2020). The first reports of MR in infants under 6 months of age used violation-of-expectation (VoE), habituation, or familiarization methods; these procedures have been used in the majority of later studies examining MR in infancy (see Moore and Johnson, 2020, for a review of work conducted in this area prior to 2021).

Moore and Johnson (2008) habituated 5-month-olds to video images of 3D objects rotating in 3D space around a vertical axis. Following the decline in visual fixation that characterizes habituation, infants typically look longer when subsequently presented with a novel stimulus; thus, more looking at a novel versus a familiar test stimulus is taken to reflect discrimination and recognition (Fantz, 1964). To test MR in infants using stimuli like those in Shepard’s seminal studies of adults (Shepard and Cooper, 1982), Moore and Johnson habituated infants to simplified Shepard-Metzler objects and tested them with those same objects as well as with novel objects that were the mirror images of the habituation objects.

In studies like this (e.g., Christodoulou et al., 2016; Constantinescu et al., 2018; Gerhard and Schwarzer, 2018; Gerhard-Samunda et al., 2021; Moore and Johnson, 2011; Schwarzer et al., 2013a, 2013b; Slone et al., 2018), infants repeatedly see a video clip of an unfamiliar, asymmetrical 3D object rotating back and forth through a limited arc around an axis. Once an infant’s looking times at this object have fallen below a pre-established habituation criterion, they are tested with video clips of the now-familiar object rotating through a previously unseen angle (i.e., the “back” side of the habituation object) or the mirror image of that object (also seen rotating through the novel angle). Thus, both of the test displays are novel—the infants have never seen either object from the perspective seen in the test trials—but one of the test stimuli represents the original habituation object, whereas the other test stimulus represents a mirror image version of that object. If infants spend significantly more time looking at the mirror image object, this is taken to mean that despite the similar appearance of the test objects, the infants can tell them apart. And if they prefer looking at the mirror image object, they must *recognize* the other object (i.e., the more familiar one, as it was the object seen during habituation), even though it is now being seen from a new perspective. As Moore, Johnson, and others have argued, this behavior plausibly indicates that the infants have been able to rotate a mental representation of at least one of the objects.

Numerous studies using this methodology have now been published. Replications of the phenomenon in 5-to 6-month-olds have been reported in Germany (Erdmann et al., 2018) and England (Constantinescu et al., 2018), and evidence has emerged that even some 3-month-olds behave as if they are capable of MR (Moore and Johnson, 2011). Other studies using similar habituation methods (Schwarzer et al., 2013a, 2013b; Slone et al., 2018) have revealed that MR performances are significantly influenced by gross motor development (i.e., crawling) and prior fine motor experience (i.e., manual exploration of objects). Likewise, VoE (Möhring and Frick, 2013; Frick and Möhring, 2013) and change detection (Lauer et al., 2015) methodologies have provided convergent evidence consistent with the conclusion that infants are capable of MR under at least some circumstances.

Nonetheless, some research teams have failed to find MR competence in infants. For example, Beckner et al. (2023b) failed in two experiments to replicate the effects that Lauer et al. (2015) observed using a change detection task. Similarly, although Erdmann et al. (2018) detected evidence of MR in 5-to 6-month-olds using a habituation task, they did not find evidence of MR in 9-to 10-month-olds. And in a study utilizing real stimulus objects, Möhring and Frick (2013) saw evidence of MR only in 6-month-olds who had been given hands-on experience with the objects; infants without such experience provided no evidence of MR.

When a collection of studies has yielded disparate results like these, meta-analysis offers a robust way to establish the direction and magnitude of an effect across those studies (Rosenthal and Rosnow, 1991). A recent meta-analysis examining the effect sizes obtained in 62 experiments on over 1,700 infants between the ages of 3 and 16 months found significant (*p* = 0.004) support for the claim that infants are capable of MR (Enge et al., 2023). Taken together, therefore, available evidence suggests that MR emerges in some infants before their first birthday.

### MR in male and female infants

A separate question is whether the sex difference observed in studies of MR in adults also characterizes infant populations. Several early studies suggested that male infants, on average, might be capable of MR, but these studies found no evidence that female infants, on average, are capable of MR (Constantinescu et al., 2018; Moore and Johnson, 2008, 2011; Quinn and Liben, 2008, 2014). That is, only male infants in these studies behaved as if they recognized a habituation object when it was being seen from a novel perspective. However, subsequent studies have yielded inconsistent results in this regard, with several yielding no sex differences (Christodoulou et al., 2016; Erdmann et al., 2018; Frick and Möhring, 2013; Gerhard and Schwarzer, 2018; Möhring and Frick, 2013; Schwarzer et al., 2013a, 2013b; Slone et al., 2018).

Given these uneven results, a meta-analysis can once again be informative. Although Enge et al.’s (2023) examination of sex differences in infants’ MR competence was not as conclusive as their examination of the existence in infancy of MR competence *per se*, this team found “that male infants recognized rotated objects slightly more reliably than female infants, [and that] this difference survives correction for small degrees of publication bias” (p. 1). Enge et al. concluded that although male infants provide more reliable evidence of MR than female infants, “this difference is small and only partially robust to publication bias” (p. 11). However, it is important to note that this team’s data indicated that there was only a small publication bias detectable in their sample of studies, and that a leave-one-out “analysis confirmed that the current results are robust to the effects of individual outlier experiments” (p. 8). Taken together, the results of this meta-analysis support the conclusion that there is a sex difference in infants’ performances on MR tasks, albeit a small one.

### The role of angle-of-rotation in studies of MR

We have argued that when infants are faced with a never-before-seen view of a familiar object and a never-before-seen view of a novel, mirror image of that object, if they prefer to look at the mirror image object, this should be considered evidence of MR competence. Nonetheless, other theorists have argued that this phenomenon might not qualify as *bona fide* evidence of MR (Levine et al., 2016), because the gold-standard signal of MR in adults has always required chronometric data showing people taking longer to mentally rotate objects through larger as opposed to smaller angles. Unfortunately, chronometric data like these have not yet proven possible to collect in studies of infants, because there is currently no way to measure the speed at which infants assess the congruence of an object with a rotated mirror-image-version of that object. Unlike older individuals who can simply tell us when they have decided if two objects are congruent, infants, by definition, are unable to provide verbal responses to our queries. After pointing out the need for chronometric data, Krüger et al. (2014) described a chronometric test that can be used in children as young as 3 years, but to date, no one to our knowledge has devised such a test for use with infants.

Thus, conclusive evidence that infants engage in MR may be difficult to obtain using current methods. Even so, as Hawkins et al. (2022) noted, infants *are* likely engaging “in some type of spatial transformation [in these kinds of tasks because it would be] difficult for infants to simply map the shape of the object seen in familiarization trials onto the shape of the objects seen in the test trials, since the familiarization and test objects were seen from different perspectives on the rotation axis” (p. 15). It follows that a task that requires recognizing a rotated habituation object in a test trial (and distinguishing it from its mirror image) will be more difficult as the object is moved through greater angles of rotation. So, mentally rotating an object through 5° of angle, for example, is easier than mentally rotating it through 30° of angle. Similarly, even if infants are not performing MR *per se* but instead are simply mapping the shape of a habituation object onto the shape of a test object using a kind of “piecemeal processing”—that is, “attending to spatially distinct components of the object and transforming parts (piece-by-piece) separately” (Hawkins et al., 2022, p. 4)—the task would likely be more difficult when the habituation and test objects are separated by larger rather than by smaller angles of rotation.^1^

Studies of infants’ MR through larger angles of rotation are important, because in the absence of data from such studies, infants’ behaviors in some tasks might best be explained with reference to perceptual discrimination failures rather than MR. For example, if an infant is familiarized with an object rotating from an initial position through 180° and is subsequently shown the same object rotating between 181° and 360°, recognizing the object as it rotates through the novel angle might not require MR at all if the infant is merely *unable to discriminate* the image seen at the 180° mark from the image seen at the 181° mark. Consequently, studies that require larger angles of MR are critical for evaluating if infants might be capable of MR (for additional discussion, see Moore and Johnson, 2020).

Given the recognized importance of angles of rotation, it is no surprise that some researchers studying young children have manipulated the angle through which MR is required, even if these studies have not yielded chronometric data. Although the number of such studies remains limited at this point, that number has grown in the last 10 years. Currently, there are several studies on toddlers in the 3-year-old range (Frick et al., 2013b; Krüger, 2018; Krüger et al., 2014; Pedrett et al., 2023), but we will focus here on studies that included infants up to 1 year of age.

### Studies exploring angle of rotation in infants’ MR

To the best of our knowledge, there has been only one study to date that attempted to bridge MR research with toddlers and MR research with infants. Beckner et al. (2023a) studied MR in children 1, 2, and 3 years of age using a single methodology. Their task involved showing children two-dimensional (2D) cartoon pictures of a giraffe on a computer screen that, when upright, would face either left or right; as such, these were mirror images of one another. After a strict training protocol that taught the children that a left-facing giraffe would approach a house on the left whereas a right-facing giraffe would approach a house on the right, images of these giraffes were presented in 12 different angles of rotation—in 15° increments from 0° to 180°—and eye-tracking was used to determine which house the children thought that giraffe would approach. Knowing which house was the correct one would require MR.

After eliminating from analysis any children unable to provide evidence that they understood the task, the median rotation angle at which 1-year-olds were successful in this task was 22.5 degrees, but eight of the sixteen 1-year-olds who were tested were successful at 30 degrees or more. Thus, using a rather complex task that required not just MR, but also associating an object (a giraffe) with a target location (a house), Beckner et al. were able to obtain evidence of MR in some 12-month-olds. Crucially, this team reported that children “were less likely to succeed as the angle of rotation increased, and the older children succeeded at higher angles of rotation than the younger children, replicating previous findings with other procedures” (p. 1).

In an early study of MR in infants, Quinn and Liben (2008) familiarized 3-to 4-month-olds with pictures of the numeral “1” (or its mirror image) rotated into seven different orientations, each of which was 45° of rotational angle away from their nearest neighbors. After presenting these pictures in a random order, the infants were shown a pair of numerals in a novel orientation; one member of the pair was the original numeral, now shown in the novel orientation, whereas the other member of the pair was the mirror image of the original numeral (also in the novel orientation). Although female infants treated the mirror image and the original numeral similarly, male infants exhibited a novelty preference, suggesting that they recognized the original numeral, even though it was being seen in a novel orientation. Quinn and Liben argued that the male infants’ novelty preferences constituted evidence of MR. To have succeeded at this task, the infants were thought to have mentally rotated the stimuli through 45° of angle.

Contrary to this finding, Möhring and Frick (2013) did not find evidence of MR in 6-month-olds who lacked prior manual experience with the stimulus objects, and Frick and Möhring (2013) did not find evidence of MR in infants who were twice as old as those tested by Quinn and Liben (2008). Relying on a VoE method, Frick and Möhring (2013) presented 8-and 10-month-olds with an asymmetrical object that was lowered behind an occluder. After the occluder was removed, either the original object or a mirror image version of that object was revealed in one of 5 different orientations (0°, 45°, 90°, 135°, or 180°). Frick and Möhring reported that the 10-month-olds, but not the 8-month-olds, looked longer at the impossible outcome (i.e., the mirror image version of original object). Furthermore, the different orientations of the objects seen in the test trials had no effect on the behavior of these infants. The discrepancy between Quinn and Liben’s results and Frick and Möhring’s results can potentially be explained by any of a large number of methodological or participant-sample differences between the two studies. For example, Quinn and Liben presented infants with static images of a 2D figure, whereas Frick and Möhring presented infants with video clips of a live experimenter moving a real object in 3D space.

To the best of our knowledge, the only other study of infants that examined specific angles of rotation was conducted by Gerhard and Schwarzer (2018), who tested 9-month-olds in a paradigm similar to that of Moore and Johnson (2008). These infants were shown a video of a simplified Shepard-Metzler object rotating in one direction through a 180° angle. After they habituated to that video, the infants saw two test videos in sequence, counterbalanced. One of the test videos showed the original habituation object whereas the other test video showed the mirror image of the habituation object; in both cases, the objects were seen rotating in the same direction as the object in the habituation video, but through a new 90° angle. Infants in one condition saw the test object in both videos rotating through the 90 degrees between 181° and 271°, so correctly recognizing the test object as the habituation object would have required only 1° of MR (because the habituation object stopped its rotation at the 180° mark and the test object began its rotation at the 181° mark).^2^ In contrast, infants in a second condition saw both test objects rotating between 234° and 324°, so correctly recognizing the test object as the habituation object would have required 54° of MR (because successful infants needed to rotate a mental representation of the object through the gap between 180° and 234°).

Gerhard and Schwarzer (2018) reported that the size of the gap between the final frame of the habituation video and the initial frame of the test video did influence the infants’ looking time behaviors as predicted, but only for infants who were experienced crawlers. Unlike the crawling infants, infants with less crawling experience spent nearly equal amounts of time looking at the mirror image and habituation test objects when they were being seen from the novel perspective. This finding is consistent with other work from the Schwarzer group, which has consistently found that experience with crawling affects MR performance in infancy (Gerhard-Samunda et al., 2021; Kelch et al., 2021; Schwarzer et al., 2013a; Schwarzer et al., 2013b). Gerhard and Schwarzer reported that when the angular difference between the habituation and test objects was larger, infants’ preferences for the mirror image object were diminished. Hence, these researchers concluded that object recognition in the 54° condition was more demanding than in the 1° condition because the former condition required MR across a larger gap angle.

### The current study

Of the approximately 15 studies that have been conducted on MR in infancy, only those just described have presented infants with stimuli representing discretely varied angles of rotation. As Gerhard and Schwarzer (2018) noted, “research involving systematically testing a possible effect of angular disparity on mental rotation performance in infants as it has been done in adults is extremely rare” (p. 47). The results reported in these studies raise several questions addressed by the current study.

First, the failure of the infants studied by Frick and Möhring (2013) coupled with the success of the crawling infants of nearly the same age that were studied by Gerhard and Schwarzer (2018) suggest that the VoE method might be less sensitive than the habituation method, motivating further tests with the latter method. Second, Gerhard and Schwarzer (2018) found that infants without much crawling experience failed in their habituation study, whereas Quinn and Liben (2014) found that similarly aged male infants —almost certainly including both crawlers and non-crawlers—succeeded in their habituation study. Finally, Quinn and Liben (2008, 2014) and Frick and Möhring (2013) used flat stimuli that were rotated around the Z axis (i.e., in a 2D plane like a clockface) whereas Gerhard and Schwarzer (2018) used a dynamic 3D stimulus rotating in depth; thus a new study including infants as young as Quinn and Liben’s but with stimuli more like Gerhard and Schwarzer’s would likely help generate a more complete portrait of MR in infancy.

The current study examined MR in 5-month-old infants using a habituation paradigm and video representations of simplified 3D Shepard-Metzler objects (and their mirror images) rotating in depth around their vertical axes. Because Gerhard and Schwarzer (2018) found evidence of MR through a 54° angle in some 9-month-olds, and because we planned to study younger infants, we constructed our stimuli so as to require MR through a smaller angle of rotation: 30°. Finally, because Quinn and Liben (2008) found novelty preferences when 45° rotations were required but only in their population of male infants, we chose a narrower angle of rotation that we thought might make the task somewhat easier, on the chance that female infants might also recognize the habituation object from a never-before-seen perspective and therefore prefer to look at the novel, mirror image object, too.

## Method

### Participants

Participants included 48 healthy, full-term 5-month-old infants [*M* = 153.65 days, *SD* = 7.32; males (*n* = 24): *M* = 153.25 days, *SD* = 7.39; females (*n* = 24): *M* = 154.04 days, *SD* = 7.39], recruited from a diverse population of new parents in the San Gabriel Valley of Southern California. This sample size was deemed appropriate because earlier studies of MR in infancy using similar stimuli and procedures proved to have enough power to reveal significant effects with samples of 40 (Moore and Johnson, 2008, 2011) and 48 (Christodoulou et al., 2016) participants, respectively. Twenty-one additional infants were observed but excluded from analysis due to fussiness (*n* = 15; coded by a trained observer as crying, irritable, or apparently uncomfortable), insufficient attention to the experimental stimuli (*n* = 3; infant rarely looked at the stimuli, looking instead at the ceiling, its parent, or its toes), sick on the test day (*n* = 2; infant vomited during the study), or a strong side bias (*n* = 1; infant only looked at the monitor on the left during the test trial). The observer was always blind to the infant’s assigned condition, and the decision to exclude the data was determined prior to seeing the data. Not counting two additional infants who were excluded due to technical malfunctions during their visit, our attrition rate was 21 out of 69, or 30%, which is not unusual in studies of young infants’ cognitive or perceptual processes (e.g., Babineau et al., 2020; He and Arunachalam, 2023; He and Lidz, 2017; Quinn et al., 2006).

### Stimuli

**Figure 1:**
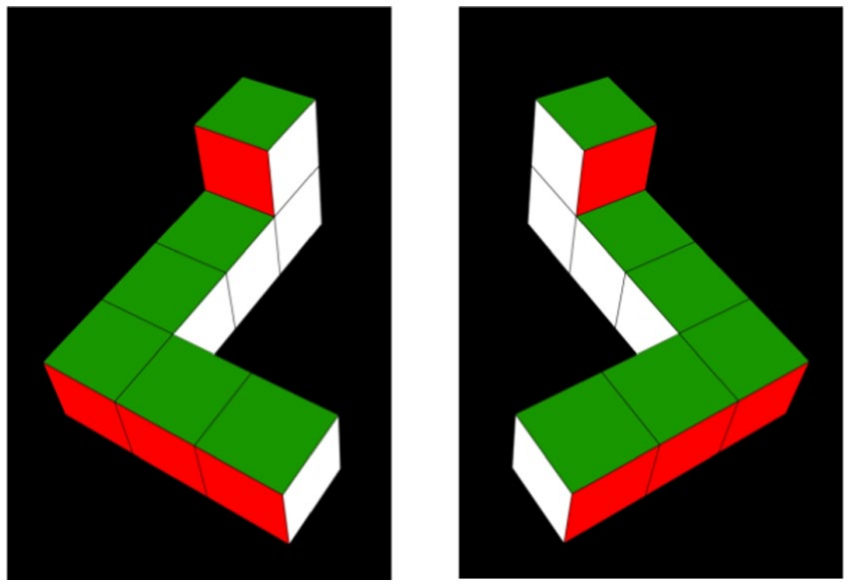
Habituation objects. Habituation objects were 2D video depictions of simplified 3D Shepard-Metzler objects (Shepard and Metzler, 1971), arbitrarily referred to as the L-object (pictured on the left in a 0°/360° orientation), and its mirror image, the R-object (pictured on the right in a 0°/360° orientation).

The stimuli consisted of four videos representing 3D, simplified Shepard-Metzler geometric block-shaped objects developed by Moore and Johnson (2008). The stimuli were displayed on a black background and rotated as described in Moore and Johnson (2008), but through different angles. The objects were arbitrarily referred to as the “L-object” and the “R-object” (see Figure 1). Each object was constructed of seven cubes attached rigidly with 90° bends at its top and bottom; a two-cube bar (*x*-axis) was attached at the bottom of a straight central bar formed of four cubes (*y*-axis), and a one-cube bar (*z*-axis) was attached to the top of this central bar. If viewed from above, all visible faces of the objects were yellow; if viewed from below, all visible faces were red. Viewed from the front, right, back, and left, the faces were purple, blue, white, and green, respectively. The L-and R-objects were mirror images of one another (as shown in Figure 1). Stimuli like these have been commonly used in studies of infants’ MR (e.g., Christodoulou et al., 2016; Constantinescu et al., 2018; Gerhard and Schwarzer, 2018; Moore and Johnson, 2008, 2011; Schwarzer et al., 2013a, 2013b; Slone et al., 2018), having been originally chosen because of their similarity to those used in Shepard’s seminal studies of MR in adults (Shepard and Cooper, 1982).

**Figure 2:**
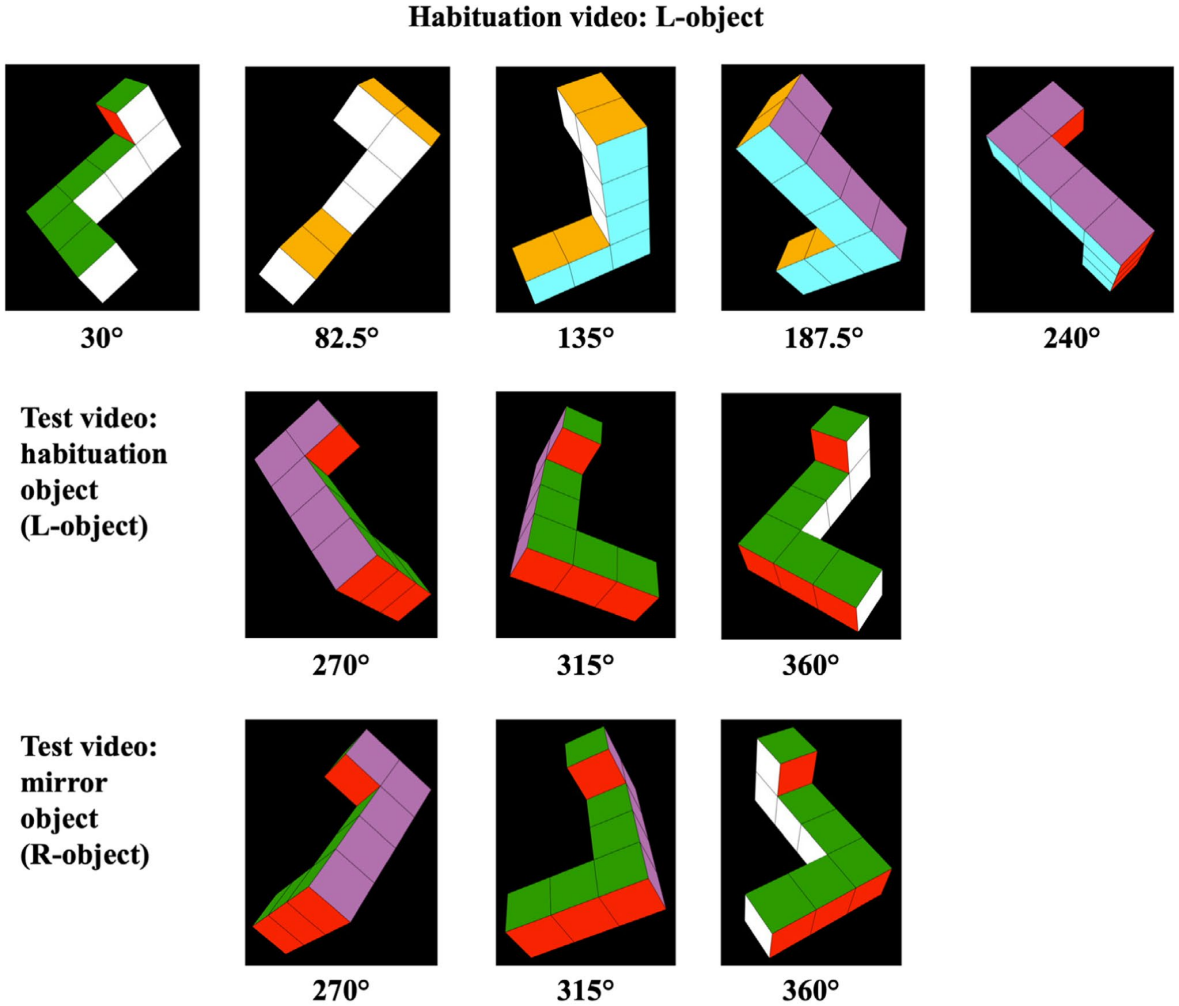
Frames sampled from habituation and test videos. The objects represented in the habituation videos rotated back and forth through a 210° angle (30° to 240°). Half of the infants saw the habituation object pictured in the top row of images here (i.e., the L-object); the other half of the infants saw a habituation video displaying the rotating R-object (not shown). The objects represented in the test videos (pictured in the bottom two rows of images here) rotated back and forth through a previously unseen 90° angle (270° to 360°/0°).

**Figure 3:**
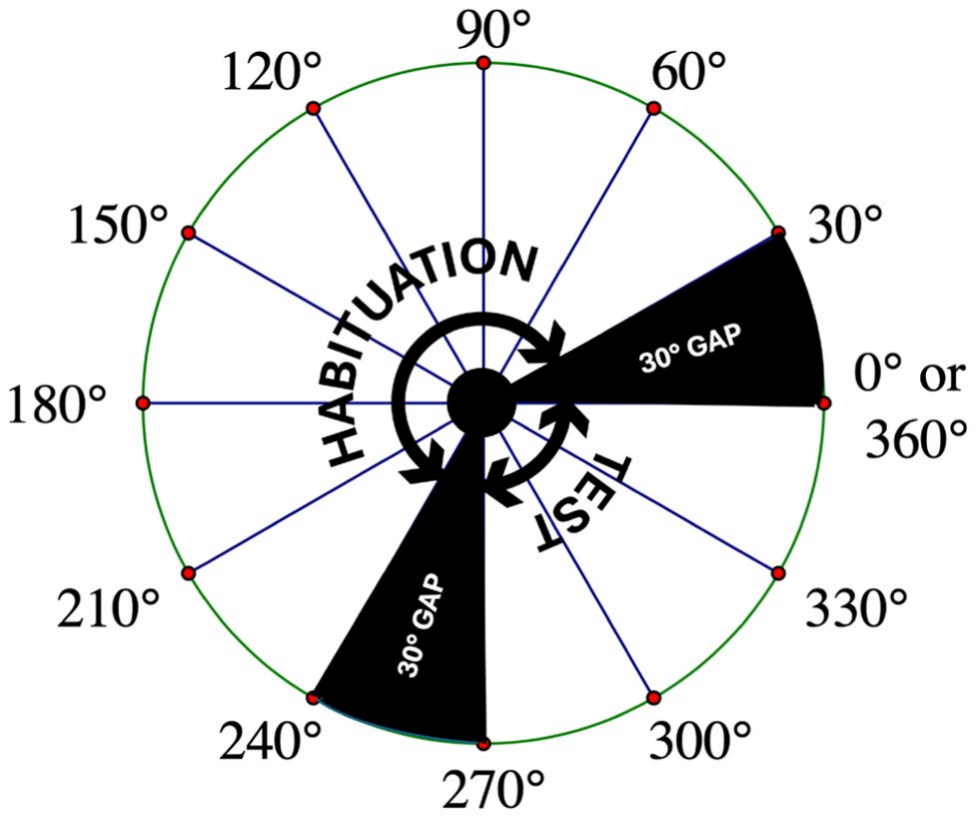
Representation of the rotational angles covered by the habituation and test stimuli. A schematic of a bird’s-eye view of the rotational angles covered by the habituation and test stimuli. Also shown are the 30-degree gaps in which the stimulus objects are never seen by the infant. To recognize the habituation object when it is presented from a novel perspective during the test trials, infants must mentally rotate a representation of the object through one of these gaps.

Each habituation video comprised 140 sequential perspective projections. Each of these projections represented the same object rotated an additional 1.5° around the vertical (Y) axis. When these videos were presented at 30 frames per second, this sequence of images appeared as an object rotating at 45° per second through a 210° arc, from 30° to 240° (see the top row of images in Figure 2). Once the object reached the maximum extent of its rotation, it reversed course and began rotating back to its starting position. Unlike the videos used by Moore and Johnson (2008), the stimulus videos left two 30° gaps between the terminal points in the habituation videos and the terminal points in the test videos. Consequently, the L-and R-objects in the *test* videos rotated through a 90° arc that commenced at 270° and terminated at 360°/0° (see Figure 3 as well as the bottom two rows of images in Figure 2). The 30° gaps meant that the objects were never seen rotating between 0° and 30° or between 240° and 270° in either the habituation or test trials. As in the habituation videos, the objects in the test videos rotated back and forth continuously between their starting position (at 270°) and the maximum extent of rotation (at 360°/0°). Aside from being mirror image objects, the L-and R-test stimuli were otherwise identical in all aspects.

### Apparatus and procedure

Upon arrival, parents and infants were greeted by a research assistant who guided them to the lab’s waiting room and provided them with informed consent documents and a questionnaire seeking information about the mother’s pregnancy, the infant’s birth, and the overall health of the infant, as well as the current state of the infant on the testing day (e.g., had the infant slept well the night before, was the infant fed and changed, etc.). Once the necessary paperwork was completed, the parent and infant were led to the testing room where a trained observer described the study procedure to the parent. To ensure optimal comfort of the infant, all infants were tested while sitting on their parent’s lap in a darkened testing room. Parents were seated 1 m away from two 53 cm monitor screens which were separated by 39 cm (from one monitor edge to the other).

As detailed in Christodoulou et al. (2016), a two-monitor experimental apparatus can be of value in some studies of infant cognition, because designs in which stimuli are presented in pairs might reduce demand on visual short-term memory relative to that required in single-monitor studies (Oakes and Ribar, 2005). In addition, this approach enables assessment of visual preferences directly, which permits inferences even when infants do not fully recover looking following habituation. Finally, although habituation studies often end with test trials that alternate between familiar and novel stimuli, a two-monitor setup allows for the simultaneous testing of interest in the two kinds of stimuli, rendering alternating test trials unnecessary.

Parents were asked to keep their eyes closed throughout the procedure. All parents complied with this instruction, meaning parents were not able to systematically influence their infants’ visual preferences. An IBM PC clone running custom software was used to present the stimuli on the monitors, time trials, calculate when the pre-established habituation criterion was met, and store data.

One of two trained observers, who was not visible to the infant and who was blind to the infant’s group assignment and to the stimuli shown, observed and coded a given infant’s behavior in both habituation and test trials using a joystick. The joystick was used both to initiate trials and to record the duration of the infant’s looks at each of the two monitors (i.e., the joystick was held to one side as long as the infant looked to that side, and the joystick was held to the other side as long as the infant looked to the other side). Given the distance between the display monitors and the infant participants’ location 1 m away from the screens, infants needed to move their eyes (and/or their heads) quite a distance when observing stimuli in the 2-monitor testing room. Accordingly, it was readily apparent when they fixated one screen instead of the other screen, as evidenced by the fact that the lab’s two trained observers’ reliability scores were consistently greater than *r* = 0.90. Reliability scores were computed by comparing looking time data that were recorded simultaneously by a trained observer and a trainee observer while pilot infants were tested in the task.

The sequence of the trials was similar to the sequence used by Christodoulou et al. (2016). The study began with infants randomly assigned to one of two habituation groups (i.e., habituation to the L-object or to the R-object). Once assigned to a group, infants observed the same habituation video on both monitors over the course of several habituation trials (i.e., either a rotating L-object or a rotating R-object appeared on both monitors simultaneously, showing identical images throughout the habituation trials). Using these two habituation groups, we were able to effectively control for any spontaneous preferences the infants might have had for these stimuli. To ensure the infants’ attention was drawn back to the video display screens before the start of each trial, an attention-getter stimulus was used on both monitors.

Every trial (including both habituation and test trials) was initiated by the observer, who pressed a button on the joystick to signify that the attention-getter stimulus had effectively drawn the infant’s attention to one of the display screens; pressing this button caused a pre-selected stimulus video to begin running on the screens. Then, using the joystick, the observer recorded infant fixations to the left and right displays in real time. Each habituation trial ended either 2 s after the observer released the joystick to indicate that the infant was no longer looking at either of the monitors, or after 60 s (whichever occurred first). If the infant’s attention to one of the displays resumed in the 2-s interval, the habituation trial continued. An infant was deemed habituated when their average time fixating the habituation stimulus (across both monitors) decreased in a four-trial block to 50% of their average fixation time in the first four habituation trials. Given this habituation criterion, each infant viewed a minimum of five habituation trials. Infants who did not habituate saw a maximum of 12 habituation trials.

Once the infant either habituated or experienced 12 habituation trials (whichever came first), they viewed a series of two test trials. Infants were randomly assigned to counterbalanced test trial groups. Infants who had been exposed to the L-habituation object (*n* = 24; males = 12, females = 12) were equally and randomly assigned to the L-object test stimulus appearing either (a) on the left screen during the first test trial (males = 6, females = 6) or (b) on the right screen during the first test trial (males = 6, females = 6). A similar random-assignment procedure was used for infants who had viewed the R-habituation object (*n* = 24; males = 12, females = 12), with the L-object test stimulus appearing either (a) on the left display during test trial one (males = 6, females = 6) or (b) on the right display during test trial one (males = 6, females = 6).

In contrast to the habituation trials during which infants viewed an identical object on both monitors, the test trials presented the habituation object (now seen from a previously unseen perspective) on one monitor, and a novel object (the mirror image of the habituation stimulus) on the other monitor. Both test stimuli were seen rotating through 90° angles, meaning that all views of the object were novel with respect to the views provided of the original habituation objects. Left–right positions of these stimuli were subsequently reversed for the second test trial. During each of the two test trials, the stimuli remained on both monitors—rotating in sync, so that at every moment, the images on the left and right monitors were mirror-images of one another—until infants accrued a total of 20 s of looking time across the two monitors.

### Statistical approach

The data were analyzed using IBM SPSS Statistics (Version 27). The preliminary statistical approach included a series of *t*-tests using infants’ habituation times and number of trials-to-habituation, consistent with previously published studies (see Christodoulou et al., 2016; Moore and Johnson, 2008, 2011). Mixed analyses of variance (ANOVAs) were subsequently conducted on the test trial data to determine if any sex differences or novelty/familiarity preferences existed in fixation of the test stimuli. In addition to including sex (female vs. male) and stimulus type (novel vs. familiar) as factors in our initial ANOVA, we included test trial (test trial 1 vs. test trial 2) as a factor, because the test stimuli were obviously more novel when seen in the first test trial than when seen again in the second test trial; as a result, infants might have behaved differently in these two test trials. Although we had no theoretically motivated hypotheses concerning this factor, we considered it important to inquire about any of its effects on the data. Finally, post-hoc *t*-tests were conducted to further examine the test trial data, exploring differences in looking at the novel and familiar test objects.

## Results

### Preliminary analyses

The primary dependent measure for the preliminary analyses was looking time during the habituation trials. Three *t*-tests were used to assess if time-to-habituation was affected by (a) the sex of the infant, (b) habituation with the L-versus the R-object, or (c) which monitor displayed the novel stimulus object in the first test trial. No statistically significant differences were found between male (*M* = 127.32 s, *SD* = 47.40) and female (*M* = 148.04 s, *SD* = 104.53) infants in terms of time-to-habituation, *t*(32.08) = −0.89, *p* = 0.383. Further, the difference between time-to-habituation for infants habituated with the L-object (*M* = 141.95 s, *SD* = 82.91) versus the R-object (*M* = 133.41 s, *SD* = 80.53) was not statistically significant, *t*(46) = 0.36, *p* = 0.719. Similarly, no statistically significant differences in times-to-habituation were found between infants who saw the novel stimulus located on the left monitor in test trial one (*M* = 147.46 s, *SD* = 94.99) and infants who saw the novel stimulus located on the right monitor in test trial one (*M* = 127.89 s, *SD* = 64.60), *t*(46) = 0.84, *p* = 0.408.

Three additional *t*-tests were used to assess if number-of-trials-to-habituation was affected by (a) the sex of the infant, (b) habituation with the L-versus the R-object, or (c) which monitor displayed the novel stimulus object in the first test trial. No statistically significant differences were found between male (*M* = 9.13 trials, *SD* = 2.66) and female (*M* = 9.04 trials, *SD* = 2.49) infants in terms of number-of-trials-to-habituation, *t*(46) = 0.11, *p* = 0.911. Similarly, the difference between the number-of-trials-to-habituation for infants habituated with the L-object (*M* = 9.17 trials, *SD* = 2.53) versus the R-object (*M* = 9.00 trials, *SD* = 2.62) was not statistically significant, *t*(46) = 0.22, *p* = 0.824. Further, no statistically significant differences in number-of-trials-to-habituation were found between infants who saw the novel stimulus located on the left monitor in test trial one (*M* = 8.96 trials, *SD* = 2.79) and infants who saw the novel stimulus located on the right monitor in test trial one (*M* = 9.21 trials, *SD* = 2.34), *t*(44.65) = −0.34, *p* = 0.738.

Finally, although 48 infants were included in the final sample, only 33 infants habituated in fewer than 12 trials; the remaining 15 infants did not successfully habituate to the habituation stimuli. A chi-square test was conducted to examine differences between male and female infants in successful habituation. This test revealed that approximately equal numbers of male (*n* = 16) and female (*n* = 17) infants successfully habituated in fewer than 12 habituation trials, 𝜒^2^(1) = 0.10, *p* = 0.755.

### Main analyses

The primary dependent measure in the main analyses was looking time during test trials at the habituated (i.e., familiar) object and the mirror image (i.e., novel) object, both seen from a new perspective. A 2 (sex: female versus male) by 2 (test stimulus fixated: novel versus familiar) by 2 (test trial: 1 versus 2) mixed ANOVA was conducted to examine these looking times. Sex was a between-subjects variable and both test stimulus and test trial were repeated-measures variables. This analysis revealed a marginally significant effect of test trial, *F*(1, 44) = 3.75, *p* = 0.059, reflecting slightly more looking, on average, in test trial 1 (*M* = 19.44 s, *SD* = 1.95) than in test trial 2 (*M* = 18.44 s, *SD* = 3.43). This effect was qualified by a 3-way interaction between sex, test stimulus fixated, and test trial, *F*(1, 44) = 3.91, *p* = 0.054.

Consequently, we next examined the data from test trial 1 and test trial 2 in separate 2 (sex: female versus male) by 2 (test stimulus fixated: novel versus familiar) mixed ANOVAs. The analysis of the test trial 1 data revealed a main effect of sex, *F*(1, 46) = 4.13, *p* = 0.048, with a medium effect size of η_p_^2^ = 0.08. The males’ average looking time at the test stimuli in test trial 1 (*M* = 10.00 s, *SD* = 4.11) was significantly greater than that of the females’ (*M* = 9.46 s, *SD* = 4.02).

**Figure 4:**
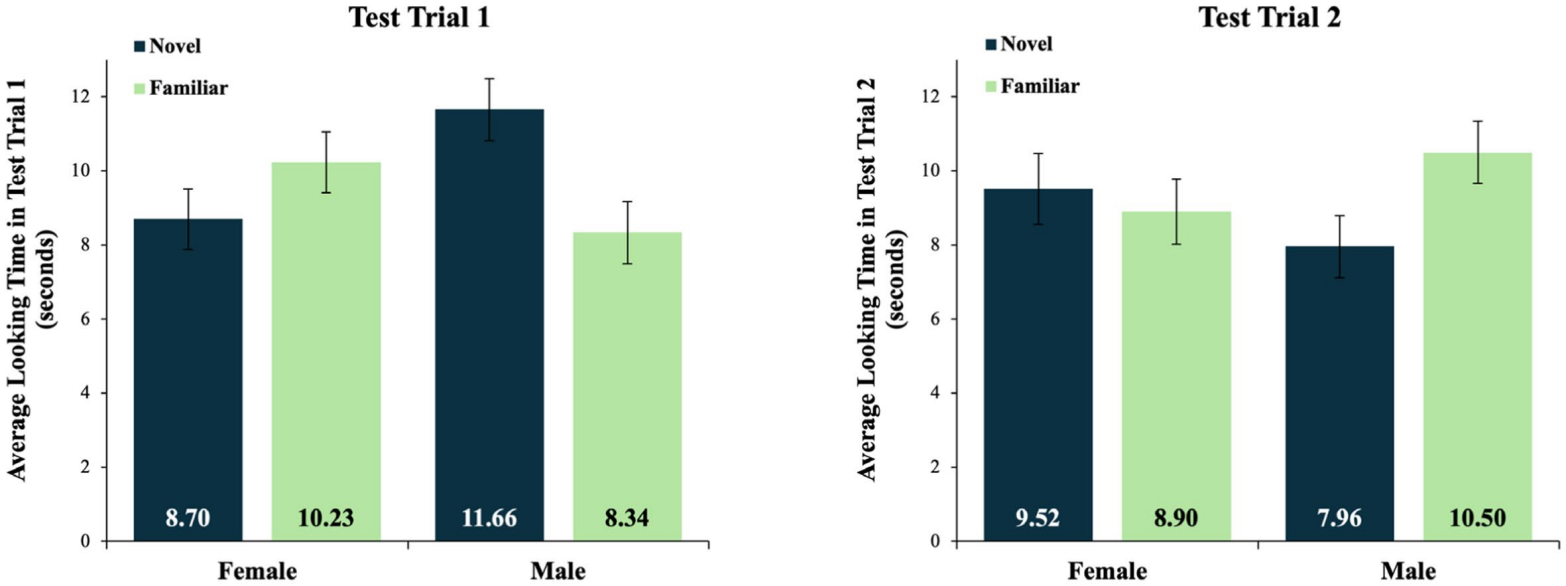
Average looking times for test trials 1 and 2. Mean looking times in test trials 1 and 2, by sex and test stimulus type. Average looking times are in seconds. The data from test trial 1 reflect a significant interaction between sex and test stimulus type. There were no significant effects in test trial 2, and no significant effects in a combined analysis of the data averaged across both test trials. Error bars indicate standard errors of the mean.

The main effect of sex in the test trial 1 data was qualified by a significant interaction between sex and test stimulus, *F*(1, 46) = 4.50, *p* = 0.039, with a medium effect size of η_p_^2^ = 0.09. Specifically, in test trial 1, male infants preferred the novel (*M*_NOV_ = 11.66 s, *SD* = 4.11) over the familiar (*M*_FAM_ = 8.34 s, *SD* = 4.11) stimulus, whereas the female infants preferred the familiar (*M*_FAM_ = 10.23 s, *SD* = 4.03) over the novel (*M*_NOV_ = 8.70 s, *SD* = 4.01) stimulus (see Figure 4).

In contrast, the analysis of the looking times in test trial 2 revealed no significant main effects or interactions. In test trial 2, the male infants looked, on average, at the novel stimulus for 7.96 s (*SD* = 4.03) and at the familiar stimulus for 10.50 s (*SD* = 4.01). The female infants looked, on average, at the novel stimulus for 9.52 s (*SD* = 4.61) and at the familiar stimulus for 8.90 s (*SD* = 4.21) (see Figure 4). A similar analysis of data averaged across test trials 1 and 2 revealed no significant main effects or interactions, as the insignificant effects in test trial 2 eroded the differences that were otherwise detectable in the data from test trial 1.

#### Looking times at the novel and familiar stimuli in test trial 1

Post-hoc comparisons of the male infants’ looking at the novel and familiar test stimuli in test trial 1 were conducted using a dependent samples *t*-test. This test revealed a marginally significant preference for the novel (*M* = 11.66 s, *SD* = 4.11) over the familiar test object (*M* = 8.34 s, *SD* = 4.11), *t*(23) = −1.98, *p* = 0.060. In contrast, the same test of the female infants’ looking in this test trial revealed more similar looking times at the novel (*M* = 8.70 s, *SD* = 4.01) and familiar (*M* = 10.23 s, *SD* = 4.03) test stimuli, *t*(23) = 0.984, *p* = 0.335. Finally, an independent samples *t*-test revealed that male infants (*M* = 11.66 s, *SD* = 4.11) looked significantly longer at the novel object in this test trial than did female infants (*M* = 8.70 s, *SD* = 4.01), *t*(46) = 2.53, *p* = 0.015.

#### Preference scores in test trial 1

Novelty preference scores in test trial 1 were calculated using the following formula: [looking time at the novel object/(looking time at the novel object + looking time at the familiar object)]. This formula yielded a percentage of total looking that was devoted to looking at the novel object in test trial 1. An independent samples *t*-test comparing the males’ and females’ novelty preference scores revealed that in this test trial, the male infants’ novelty preference scores (*M* = 0.58 novelty preference, *SD* = 0.21) were significantly great than the female infants’ novelty preference scores (*M* = 0.46 novelty preference, *SD* = 0.20), *t*(46) = 2.15, *p* = 0.037. Even so, the male infants’ novelty preference scores were only marginally greater than chance (i.e., a test value of 0.50, that is, no novelty or familiarity preference), *t*(23) = 1.98, *p* = 0.060. In contrast, female infants’ novelty preference scores were not different from chance, *t*(23) = −1.05, *p* = 0.306.

## Discussion

The current study was designed to explore the hypothesis that young infants tested in a habituation study would be able to mentally rotate a 3D representation of an object in depth through a 30° angle. Although reaction-time data are considered to provide the best evidence of MR, such data have not yet proven possible to collect from infants, so studies that explore infants’ MR of an object through one or more specific angles represent the state of the art. To date, a small number of studies like this have been conducted, but most of them have examined the behavior of older infants. Beckner et al. (2023a), for example, found that half of the 1-year-olds they tested were able to succeed in their MR task and Frick and Möhring (2013) also reported evidence of MR, but not in infants less than 10 months old. Quinn and Liben (2008) found evidence of MR through a 45° angle in male 3-to 4-month-olds, but only using exceptionally simple 2D stimuli that did not rotate in depth. The published study most similar to the current study was conducted by Gerhard and Schwarzer (2018), who found that 9-month-olds’ MR performance *was* influenced by angles-of-rotation, but only for experienced crawlers. Thus, the current study was the first test of younger infants’ ability to succeed in a test requiring MR of a relatively complex 3D object through a 30° angle in 3D space.

It is common in studies using a habituation design to provide infants with two test trials, because the final trial before an infant reaches a habituation criterion yields a low level of looking *by design*, and as a result, the first test trial is likely to yield *increased* looking, not because the infant is more interested in the stimulus presented in that trial, but because of regression to the mean (Oakes, 2010). For this reason, our labs provide infants with at least two test trials in all of our habituation studies. However, in the current study, test trial 2 was not essential, because the use of two monitors displaying two different test stimuli meant that recovery of looking after habituation was not the response of interest; instead, what mattered was which of the two stimulus objects the infants spent more time fixating. Thus, the fact that significant results emerged only in test trial 1 was not particularly surprising; by the time the infants saw test trial 2, the test stimuli they saw were no longer as compelling as they were in test trial 1. Therefore, the following discussion refers only to the data collected in test trial 1.

The main analyses of these data revealed a significant interaction between sex and test stimulus, such that male infants had a nominal preference for the novel over the familiar test stimulus, whereas female infants had a nominal preference for the familiar over the novel test stimulus. The effect size of this interaction was not small. Post-hoc *t*-tests indicated that male infants looked significantly longer at the novel object than female infants did, and that their novelty preference scores were significantly greater than female infants’ novelty preference scores.

Despite these significant results, post-hoc tests revealed that when the male infants’ data were considered alone, their novelty preference scores did not quite reach the *p* < 0.05 level of significance; male infants had a marginal preference for the novel over the familiar test stimuli. In part, these marginal results reflect our decision to conduct two-tailed *t*-tests; although the one-tailed tests we would have conducted if we were confident of finding novelty rather than familiarity preferences would have been significant at the *p* < 0.03 level, two-tailed tests were required because studies of MR in infants sometimes find familiarity preferences (Christodoulou et al., 2016; Erdmann et al., 2018; Gerhard and Schwarzer, 2018; Moore and Johnson, 2011; Schwarzer et al., 2013b). In addition, because male infants constituted only half of our participant population (*n* = 24), it is possible that tests of effects in this group were underpowered.

Thus, although this study does not offer conclusive evidence that young male infants can mentally rotate a 3D representation of an object in depth through a 30° angle, the results are consistent with published data indicating that male 5-month-olds, on average, are capable of MR (e.g., Constantinescu et al., 2018; Moore and Johnson, 2008, 2011; Quinn and Liben, 2008, 2014). In contrast, the female 5-month-olds we tested looked at the two test stimuli for similar amounts of time, suggesting that they might not have recognized the object seen during the habituation trials when it was presented in a novel orientation during the test trials.

As Levine et al. (2016) and Moore and Johnson (2020) have noted, the lack of evidence that female 5-month-olds are capable of MR cannot be taken as evidence that these infants are *not* capable of MR. Levine et al. correctly pointed out that “there are many reasons why infants may not look longer at the novel mirror image stimulus … they may find both test stimuli interesting—after all, both are presented [from a perspective] that was not seen during the habituation trials. … This possibility would be consistent with a sex difference, but not one that reflects an ability of male but not female infants to mentally rotate figures” (pp. 5–6). Thus, although our current findings are consistent with Enge et al.’s (2023) meta-analytic conclusion that male infants are more likely than female infants to provide evidence of MR competence, these findings should not be considered evidence of MR *in*ability on the part of female infants.

One outstanding question is why studies of MR in infants only *sometimes* find sex differences. A separate question is why a sex difference might exist, if in fact it does. Regarding the first question, discrepant results in this research area are perhaps to be expected given the large number of differences in the participant populations studied and the methodologies deployed by researchers working in this area. Actually, any of the differences that could account for significant versus null results *in general* in infant MR studies could also potentially explain why some studies do and some studies do not find sex differences. For example, Möhring and Frick (2013) suggested that differential performances across studies could reflect the amount of rotation that participants see the stimuli undergoing prior to their test. Beckner et al. (2023b) considered the possibility that their failure to replicate Lauer et al. (2015) might have resulted from the slight differences in viewing distances that characterized these two studies. In addition, Beckner et al. tested their infants during the COVID-19 pandemic, so infants were tested online using a web-based system, whereas Lauer et al. tested their participants live, in a physical laboratory. Although Erdmann et al. (2018) detected MR in their young infants, they failed to find such evidence in older infants, a difference that could perhaps be because the stimuli were too boring for 9-to 10-month-old infants. These ideas (and others) might explain why some studies find evidence of MR in infants whereas others do not, and similar kinds of differences across studies could be invoked to explain why sex differences sometimes are and sometimes are not detected, too. However, all of these ideas remain mere speculation at this point.

Likewise, it is not yet clear why sex differences on the current MR task might exist. Constantinescu et al. (2018) reported that early postnatal testosterone exposure and parental attitudes about gender were both related to infants’ MR performances, suggesting two possible answers to this question. However, testosterone and parental attitudes have a very large number of knock-on effects—testosterone, for example, induces axonal and dendritic growth, influences cell numbers in certain brain structures, regulates cell death, supports synaptogenesis, and affects neural connectivity (Hines, 2011; Moore, 2012)—so it is neither surprising nor especially enlightening to identify them as factors in early behavioral sex differences. These kinds of factors likely play roles in the development of many (if not all) of the early sex differences that have been observed, including, for example, the findings that male and female infants differ in their activity levels (Campbell and Eaton, 1999; Oller et al., 2023), their visual preferences for groups of children versus individual children (Benenson et al., 2007), their fine and gross motor activity (Piek et al., 2002), and their sensitivity to painful stimuli (Guinsburg et al., 2000). Other established sex differences that might intuitively be thought to influence behavior in the current MR task include infant boys’ greater visual contrast sensitivity (Dobkins et al., 2009), earlier-maturing visual accommodative responses (Horwood and Riddell, 2008), and better performances on event-mapping tasks (Alexander and Wilcox, 2012) relative to infant girls. The large array of sex differences in brain development, sensation, perception, and cognition makes it impossible at present to offer a precise explanation as to why male and female infants responded differently in our MR task. Just as we can only speculate about why sex differences sometimes are and sometimes are not detected in infant MR tasks, we can currently only speculate about why sex differences might exist in infants’ MR competence.

## Conclusion

Given that our stimuli were significantly more complex than those used by Quinn and Liben (2008, 2014) and that Gerhard and Schwarzer (2018) found evidence of MR through a 54° angle only in *crawling* 9-month-olds, it was not clear prior to our study that 5-month-olds would be able to provide evidence of MR in the current task. After all, none of the infants we tested would have had experience crawling, and they were all quite a bit younger than the infants tested by Gerhard and Schwarzer (2018). This could be why the male infants’ novelty preferences in the current study were only marginally significant. Regardless, our male infants’ nominal preferences for the mirror image test stimuli suggest that they might have been performing MR through a 30° angle. Otherwise, it would be difficult to explain why they would have had such a marked preference for the mirror image test stimulus, since it looked very much like the never-before-seen view of the other test stimulus. Both test stimuli were novel, so the only driver of a preference could have been a *recognition* that the “back side” view of the habituation object was still a view of that familiar object, whereas the very similar view of the mirror image object was a view of a genuinely novel object. Such recognition would likely signal mental rotation of an object, in this case through a challenging 30° angle. The relatively large size of this angle considerably reduces concern that the male infants’ behaviors reflected a mere *inability to discriminate* the images seen in the frames at the extreme ends of the habituation and test videos, and instead adds to the existing body of research suggesting that many of these infants might be capable of genuine MR.

Because the reaction-time data that offer the strongest evidence of MR cannot (yet) be collected from infants, conclusive proof of MR in young infants remains elusive. Consequently, some researchers have argued that the methods used in the current study are not suitable for *confirming* that infants can engage in analog MR processes (e.g., Hawkins et al., 2022). We agree. Nevertheless, studies that vary angles of rotation have the potential to improve our confidence that infants are capable of MR, as such studies at least have the potential to reveal “the classic signature pattern of mental rotation—more difficulty on stimuli with greater angular disparities” (Levine et al., 2016, p. 5). Thus, innovative methods that vary angles of rotation, like the method pioneered by Beckner et al. (2023a) with older infants, appear to be promising avenues for additional research, particularly if they prove implementable with infants younger than 1 year of age.

The emerging consensus that some infants become capable of MR—or at very least, some type of spatial-cognitive transformation—before they turn 6 months of age has some important implications. Because this sort of spatial cognition is of practical utility in a wide variety of tasks, it would be useful to understand how it develops. In addition, because MR competence has proven amenable to training in older populations (Baenninger and Newcombe, 1989; Cherney et al., 2003; Fernández-Méndez et al., 2018; de Acedo Lizarraga and García Ganuza, 2003), it is possible that some kinds of early interventions might be of value in the case of individuals who have not yet mastered this kind of skill. Although a sex difference in MR performance in young infants is more controversial than the presence of the competence *per se*, the current study provided additional evidence that female and male infants behave differently, on average, in this kind of MR task. To date, few studies have examined how MR competence comes to emerge during the first 5 months of postnatal development, although Constantinescu et al.’s (2018) data suggest that both hormonal and parental-attitudinal factors might play important roles. Moreover, research on developmental changes in the frontal cortex and the development of attentional control in infancy could provide more insight into the mechanisms underlying MR skills (Ellis et al., 2021; Reynolds and Romano, 2016; Yang et al., 2020). Future research in this area should work to illuminate the mechanisms that lead to the emergence of this competence early in development.

## Data Availability

The raw data supporting the conclusions of this article will be made available by the authors, without undue reservation.

## Footnotes

^1^Hawkins et al. (2022) presented data that suggested that the infants tested in their paradigm were not “engaged in analog spatial transformation of the test objects that preserved the spatial relation between object parts” (p. 15). That is, Hawkins et al. argued that rather than engaging in genuine MR, the infants they tested were engaging in another kind of spatial transformation requiring “piecemeal processing of object parts” (p. 15). However, we think it is highly unlikely that the infants in our study could have been relying on a piecemeal processing strategy, because such a strategy would require a working memory capacity that does not characterize infants younger than 6 months of age (Reynolds and Romano, 2016; Ross-Sheehy et al., 2003).

^2^Alternatively, as discussed previously, successful infants could have treated the familiar object seen from the “novel” perspective as familiar because of a failure to perceptually discriminate between the views at 180° and 181°.

## References

[ref1] Alexander G. M. Wilcox T. (2012). Sex differences in early infancy. Child Dev. Perspect. 6, 400–406. doi: 10.1111/j.1750-8606.2012.00247.x

[ref2] Babineau M. Shi R. Christophe A. (2020). 14-month-olds exploit verbs’ syntactic contexts to build expectations about novel words. Infancy 25, 719–733. doi: 10.1111/infa.12354, PMID: 32857439

[ref3] Baenninger M. Newcombe N. (1989). The role of experience in spatial test performance: a meta-analysis. Sex Roles 20, 327–344.

[ref4] Beckner A. G. Katz M. Tompkins D. N. Voss A. T. Winebrake D. LoBue V.. (2023a). A novel approach to assessing infant and child mental rotation. J. Intelligence 11:168. doi: 10.3390/jintelligence11080168, PMID: 37623551 PMC10455586

[ref5] Beckner A. G. Voss A. T. Phillips L. King K. Casasola M. Oakes L. M. (2023b). An investigation of mental rotation in infancy using change detection. Infant Behav. Dev. 71:101834. doi: 10.1016/j.infbeh.2023.101834, PMID: 37080014

[ref6] Benenson J. F. Markovits H. Muller I. Challen A. Carder H. P. (2007). Explaining sex differences in infants’ preferences for groups. Infant Behav. Dev. 30, 587–595. doi: 10.1016/j.infbeh.2007.03.010, PMID: 17399792

[ref7] Campbell D. W. Eaton W. O. (1999). Sex differences in the activity level of infants. Infant Child Dev. 8, 1–17.

[ref8] Casey B. M. Pezaris E. Fineman B. Pollock A. Demers L. Dearing E. (2015). A longitudinal analysis of early spatial skills compared to arithmetic and verbal skills as predictors of fifth-grade girls' math reasoning. Learn. Individ. Differ. 40, 90–100. doi: 10.1016/j.lindif.2015.03.028

[ref9] Cherney I. D. Jagarlamudi K. Lawrence E. Shimabuku N. (2003). Experiential factors in sex differences on mental rotation. Percept. Mot. Skills 96, 1062–1070. doi: 10.2466/pms.2003.96.3c.1062, PMID: 12929758

[ref10] Christodoulou J. Johnson S. P. Moore D. M. Moore D. S. (2016). Seeing double: five-month-olds’ mental rotation of dynamic, 3D block stimuli presented on dual monitors. Infant Behav. Dev. 45, 64–70. doi: 10.1016/j.infbeh.2016.09.005, PMID: 27744109

[ref11] Collaer M. L. Hines M. (1995). Human behavioral sex differences: a role for gonadal hormones during development? Psychol. Bull. 118, 55–107, PMID: 7644606 10.1037/0033-2909.118.1.55

[ref12] Conrad J. Shah A. H. Divino C. M. Schluender S. Gurland B. Shlasko E.. (2006). The role of mental rotation and memory scanning on the performance of laparoscopic skills: a study on the effect of camera rotational angle. Surg. Endosc. Other Interv. Tech. 20, 504–510. doi: 10.1007/s00464-005-0363-7, PMID: 16437266

[ref13] Constantinescu M. Moore D. S. Johnson S. P. Hines M. (2018). Early contributions to infants’ mental rotation abilities. Dev. Sci. 21:e12613. doi: 10.1111/desc.12613, PMID: 29143410

[ref14] de Acedo Lizarraga M. L. S. García Ganuza J. M. (2003). Improvement of mental rotation in girls and boys. Sex Roles 49, 277–286. doi: 10.1023/A:1024656408294

[ref15] Dobkins K. R. Bosworth R. G. McCleery J. P. (2009). Effects of gestational length, gender, postnatal age, and birth order on visual contrast sensitivity in infants. J. Vis. 19, 1–21. doi: 10.1167/9.10.19 PMC2951975 19810800

[ref16] Ellis C. T. Skalaban L. J. Yates T. S. Turk-Browne N. B. (2021). Attention recruits frontal cortex in human infants. PNAS 118:e2021474118. doi: 10.1073/pnas.2021474118 33727420 PMC7999871

[ref17] Enge A. Kapoor S. Kieslinger A. Skeide M. A. (2023). A meta-analysis of mental rotation in the first years of life. Dev. Sci. 26:e13381. doi: 10.1111/desc.13381, PMID: 36843394

[ref18] Erdmann K. Kavšek M. Heil M. (2018). Infants’ looking times in a dynamic mental rotation task: clarifying inconsistent results. Cogn. Dev. 48, 279–285. doi: 10.1016/j.cogdev.2018.10.002

[ref19] Estes D. (1998). Young children’s awareness of their mental activity. The case of mental rotation. Child Dev. 69, 1345–1360, PMID: 9839420

[ref20] Fantz R. L. (1964). Visual experience in infants: decreased attention to familiar patterns relative to novel ones. Science 146, 668–670, PMID: 14191712 10.1126/science.146.3644.668

[ref21] Fernández-Méndez L. M. Contreras M. J. Elosúa M. R. (2018). From what age is mental rotation training effective? Differences in preschool age but not in sex. Front. Psychol. 9:753. doi: 10.3389/fpsyg.2018.00753, PMID: 29867698 PMC5964331

[ref22] Frick A. (2019). Spatial transformation abilities and their relation to later mathematics performance. Psychol. Res. 83, 1465–1484. doi: 10.1007/s00426-018-1008-5, PMID: 29637258

[ref23] Frick A. Ferrara K. Newcombe N. S. (2013a). Using a touch screen paradigm to assess the development of mental rotation between 3½ and 5½ years of age. Cogn. Process. 14, 117–127. doi: 10.1007/s10339-012-0534-0, PMID: 23306783

[ref24] Frick A. Hansen M. A. Newcombe N. S. (2013b). Development of mental rotation in 3-to 5-year-old children. Cogn. Dev. 28, 386–399. doi: 10.1016/j.cogdev.2013.06.002

[ref25] Frick A. Möhring W. (2013). Mental object rotation and motor development in 8-and 10-month-old infants. J. Exp. Child Psychol. 115, 708–720. doi: 10.1016/j.jecp.2013.04.001, PMID: 23708734

[ref26] Geer E. A. Quinn J. M. Ganley C. M. (2019). Relations between spatial skills and math performance in elementary school children: a longitudinal investigation. Dev. Psychol. 55, 637–652. doi: 10.1037/dev0000649, PMID: 30550325

[ref27] Gerhard T. M. Schwarzer G. (2018). Impact of rotation angle on crawling and non-crawling 9-month-old infants’ mental rotation ability. J. Exp. Child Psychol. 170, 45–56. doi: 10.1016/j.jecp.2018.01.001, PMID: 29407187

[ref28] Gerhard-Samunda T. M. Jovanovic B. Schwarzer G. (2021). Role of manually-generated visual cues in crawling and non-crawling 9-month-old infants’ mental rotation. Cogn. Dev. 59:101053. doi: 10.1016/j.cogdev.2021.101053

[ref29] Guinsburg R. de Araújo Peres C. Branco de Almeida M. F. De Cássia Xavier Balda R. Cássia Berenguel R. Tonelotto J.. (2000). Differences in pain expression between male and female newborn infants. Pain 85, 127–133, PMID: 10692611 10.1016/s0304-3959(99)00258-4

[ref30] Hawkins L. Nyman T. M. Wilcox T. (2022). Infant's recognition of three-dimensional form: Mirror image and structurally distinct objects. Infant Child Dev. 31:e2299. doi: 10.1002/icd.2299

[ref31] He A. X. Arunachalam S. (2023). Event end-state encoding in 13-month-olds: completed and non-completed events are different. Lang. Cogn., 1–15. doi: 10.1017/langcog.2023.54

[ref32] He A. X. Lidz J. (2017). Verb learning in 14-and 18-month-old English-learning infants. Lang. Learn. Dev. 13, 335–356. doi: 10.1080/15475441.2017.1285238

[ref33] Hespos S. J. Rochat P. (1997). Dynamic mental representation in infancy. Cognition 64, 153–188, PMID: 9385869 10.1016/s0010-0277(97)00029-2

[ref34] Hines M. (2011). Gender development and the human brain. Annu. Rev. Neurosci. 34, 69–88. doi: 10.1146/annurev-neuro-061010-113654, PMID: 21438685

[ref35] Horwood A. M. Riddell P. M. (2008). Gender differences in early accommodation and vergence development. Ophthalmic Physiol. Opt. 28, 115–126. doi: 10.1111/j.1475-1313.2008.00547.x, PMID: 18339042

[ref36] Iachini T. Ruggiero G. Bartolo A. Rapuano M. Ruotolo F. (2019). The effect of body-related stimuli on mental rotation in children, young and elderly adults. Sci. Rep. 9:1169. doi: 10.1038/s41598-018-37729-7 30718610 PMC6362092

[ref37] Kail R. (1991). Controlled and automatic processing during mental rotation. J. Exp. Child Psychol. 51, 337–347. 2072082 10.1016/0022-0965(91)90081-3

[ref38] Kail R. Pellegrino J. Carter P. (1980). Developmental changes in mental rotation. J. Exp. Child Psychol. 29, 102–116, PMID: 7354259 10.1016/0022-0965(80)90094-6

[ref39] Kelch A. Schwarzer G. Gehb G. Jovanovic B. (2021). How 9-month-old crawling infants profit from visual-manual rotations in a mental rotation task. Infant Behav. Dev. 65:101642. doi: 10.1016/j.infbeh.2021.101642, PMID: 34509099

[ref40] Kerkman D. D. Wise J. C. Harwood E. A. (2000). Impossible “mental rotation” problems: a mismeasure of women's spatial abilities? Learn. Individ. Differ. 12, 253–269.

[ref41] Krüger M. (2018). Three-year-olds solved a mental rotation task above chance level, but no linear relation concerning reaction time and angular disparity presented itself. Front. Psychol. 9:1796. doi: 10.3389/fpsyg.2018.01796, PMID: 30337894 PMC6180201

[ref42] Krüger M. Kaiser M. Mahler K. Bartels W. Krist H. (2014). Analogue mental transformations in 3-year-olds: introducing a new mental rotation paradigm suitable for young children. Infant Child Dev. 23, 123–138. doi: 10.1002/icd.1815

[ref43] Lauer J. E. Lourenco S. F. (2016). Spatial processing in infancy predicts both spatial and mathematical aptitude in childhood. Psychol. Sci. 27, 1291–1298. doi: 10.1177/0956797616655977, PMID: 27528464

[ref44] Lauer J. E. Udelson H. B. Jeon S. O. Lourenco S. F. (2015). An early sex difference in the relation between mental rotation and object preference. Front. Psychol. 6:558. doi: 10.3389/fpsyg.2015.00558, PMID: 26005426 PMC4424807

[ref45] Levine S. C. Foley A. Lourenco S. Ehrlich S. Ratliff K. (2016). Sex differences in spatial cognition: advancing the conversation. WIREs Cogn. Sci. 7, 127–155. doi: 10.1002/wcs.1380, PMID: 26825049

[ref46] Levine S. C. Huttenlocher J. Taylor A. Langrock A. (1999). Early sex differences in spatial skill. Dev. Psychol. 35, 940–949, PMID: 10442863 10.1037//0012-1649.35.4.940

[ref47] Linn M. C. Petersen A. C. (1985). Emergence and characterization of sex differences in spatial ability: a meta-analysis. Child Dev. 56, 1479–1498., PMID: 4075870

[ref1002] Marmor G. S. (1975). Development of kinetic images: when does the child first represent movement in mental images? Cogn. Psychol. 9, 548–559., PMID: 30337894

[ref48] Möhring W. Frick A. (2013). Touching up mental rotation: effects of manual experience on 6-month-old infants' mental object rotation. Child Dev. 84, 1554–1565. doi: 10.1111/cdev.12065, PMID: 23432700

[ref49] Moore D. S. (2012). Sex differences in normal fetuses and infants: a commentary. Child Dev. Perspect. 6, 414–416. doi: 10.1111/j.1750-8606.2012.00258.x

[ref50] Moore D. S. Johnson S. P. (2008). Mental rotation in human infants: a sex difference. Psychol. Sci. 19, 1063–1066. doi: 10.1111/j.1467-9280.2008.02200.x 19076473 PMC2651884

[ref51] Moore D. S. Johnson S. P. (2011). Mental rotation of dynamic, three-dimensional stimuli by 3-month-old infants. Infancy 16, 435–445. doi: 10.1111/j.1532-7078.2010.00058.x, PMID: 26312057 PMC4547474

[ref52] Moore D. S. Johnson S. P. (2020). The development of mental rotation ability across the first year after birth. Adv. Child Dev. Behav. 58, 1–33. doi: 10.1016/bs.acdb.2020.01.001, PMID: 32169193

[ref53] Newcombe N. S. Booth J. L. Gunderson E. (2019). “Spatial skills, reasoning, and mathematics” in Cambridge handbook of cognition and education. eds. Dunlosky J. Rawson K. A. (Cambridge: Cambridge University Press), 100–123.

[ref54] Oakes L. M. (2010). Using habituation of looking time to assess mental processes in infancy. J. Cogn. Dev. 11, 255–268. doi: 10.1080/15248371003699977 20730029 PMC2922773

[ref55] Oakes L. M. Ribar R. J. (2005). A comparison of infants’ categorization in paired and successive presentation familiarization tasks. Infancy 7, 85–98. doi: 10.1207/s15327078in0701_7, PMID: 33430540

[ref56] Oller D. K. Gilkerson J. Richards J. A. Hannon S. Griebel U. Bowman D. D.. (2023). Sex differences in infant vocalization and the origin of language. iScience 26:106884. doi: 10.1016/j.isci.2023.106884 37378320 PMC10291326

[ref57] Pedrett S. Chavaillaz A. Frick A. (2023). Age-related changes in how 3.5-to 5.5-year-olds observe and imagine rotational object motion. Spat. Cogn. Comput. 23, 83–111. doi: 10.1080/13875868.2022.2095276

[ref58] Pedrett S. Kaspar L. Frick A. (2020). Understanding of object rotation between two and three years of age. Dev. Psychol. 56, 261–274. doi: 10.1037/dev0000871, PMID: 31804097

[ref59] Piek J. P. Gasson N. Barrett N. Case I. (2002). Limb and gender differences in the development of coordination in early infancy. Hum. Mov. Sci. 21, 621–639. doi: 10.1016/s0167-9457(02)00172-0, PMID: 12620715

[ref60] Quaiser-Pohl C. Rohe A. M. Amberger T. (2010). The solution strategy as an indicator of the developmental stage of preschool children’s mental-rotation ability. J. Individ. Differ. 31, 95–100. doi: 10.1027/1614-0001/a000017

[ref61] Quinn P. C. Liben L. S. (2008). A sex difference in mental rotation in young infants. Psychol. Sci. 19, 1067–1070. doi: 10.1111/j.1467-9280.2008.02201.x 19076474

[ref62] Quinn P. C. Liben L. S. (2014). A sex difference in mental rotation in young infants: convergent evidence. Infancy 19, 103–116. doi: 10.1111/infa.12033 19076474

[ref63] Quinn P. C. Westerlund A. Nelson C. A. (2006). Neural markers of categorization in 6-month-old infants. Psychol. Sci. 17, 59–66. doi: 10.1111/j.1467-9280.2005.01665.x, PMID: 16371145

[ref64] Reynolds G. D. Romano A. C. (2016). The development of attention systems and working memory in infancy. Front. Syst. Neurosci. 10:15. doi: 10.3389/fnsys.2016.00015 26973473 PMC4776056

[ref65] Rochat P. Hespos S. J. (1996). Tracking and anticipation of invisible spatial transformations by 4-to 8-month-old infants. Cogn. Dev. 11, 3–17.

[ref66] Rosenthal R. Rosnow R. L. (1991). Essentials of behavioral research: Methods and data analysis. New York, NY: McGraw-Hill.

[ref67] Ross-Sheehy S. Oakes L. M. Luck S. J. (2003). The development of visual short-term memory capacity in infants. Child Dev. 74, 1807–1822. doi: 10.1046/j.1467-8624.2003.00639.x, PMID: 14669897

[ref68] Rusiak P. Lachmann T. Jaskowski P. van Leeuwen C. (2007). Mental rotation of letters and shapes in developmental dyslexia. Perception 36, 617–631. doi: 10.1068/p5644, PMID: 17564205

[ref69] Rüsseler J. Scholz J. Jordan K. Quaiser-Pohl C. (2005). Mental rotation of letters, pictures, and three-dimensional objects in German dyslexic children. Child Neuropsychol. 11, 497–512. doi: 10.1080/09297040490920168, PMID: 16306024

[ref70] Schöning S. Engelien A. Kugel H. Schäfer S. Schiffbauer H. Zwitserlood P.. (2007). Functional anatomy of visuo-spatial working memory during mental rotation is influenced by sex, menstrual cycle, and sex steroid hormones. Neuropsychologia 45, 3203–3214. doi: 10.1016/j.neuropsychologia.2007.06.011, PMID: 17689571

[ref71] Schwarzer G. Freitag C. Buckel R. Lofruthe A. (2013a). Crawling is associated with mental rotation ability by 9-month-old infants. Infancy 18, 432–441. doi: 10.1111/j.1532-7078.2012.00132.x

[ref72] Schwarzer G. Freitag C. Schum N. (2013b). How crawling and manual object exploration are related to the mental rotation abilities of 9-month-old infants. Front. Psychol. 4:97. doi: 10.3389/fpsyg.2013.00097, PMID: 23459565 PMC3586719

[ref73] Shea D. L. Lubinski D. Benbow C. P. (2001). Importance of assessing spatial ability in intellectually talented young adolescents: a 20-year longitudinal study. J. Educ. Psychol. 93, 604–614. doi: 10.1037/0022-0663.93.3.604

[ref74] Shepard R. N. (1978). The mental image. Am. Psychol. 33, 125–137.

[ref75] Shepard R. N. Cooper L. A. (1982). Mental images and their transformations. Cambridge, MA: MIT Press.

[ref76] Shepard R. N. Metzler J. (1971). Mental rotation of three-dimensional objects. Science 171, 701–703, PMID: 5540314 10.1126/science.171.3972.701

[ref77] Slone L. K. Moore D. S. Johnson S. P. (2018). Object exploration facilitates 4-month-olds' mental rotation performance. PLoS One 13:e0200468. doi: 10.1371/journal.pone.0200468, PMID: 30091988 PMC6084896

[ref78] Titze C. Jansen P. Heil M. (2010). Mental rotation performance in fourth graders: no effects of gender beliefs (yet?). Learn. Individ. Differ. 20, 459–463. doi: 10.1016/j.lindif.2010.04.003

[ref79] van Tetering M. van der Donk M. de Groot R. H. M. Jolles J. (2019). Sex differences in the performance of 7–12 year olds on a mental rotation task and the relation with arithmetic performance. Front. Psychol. 10:107. doi: 10.3389/fpsyg.2019.00107, PMID: 30761050 PMC6364576

[ref80] Verdine B. N. Golinkoff R. M. Hirsh-Pasek K. Newcombe N. S. (2017). Links between spatial and mathematical skills across the preschool years. Monogr. Soc. Res. Child Dev. 82, 1–126. doi: 10.1111/mono.12280 28181246

[ref81] Voyer D. Voyer S. Bryden M. P. (1995). Magnitude of sex differences in spatial abilities: a meta-analysis and consideration of critical variables. Psychol. Bull. 117, 250–270, PMID: 7724690 10.1037/0033-2909.117.2.250

[ref82] Wai J. Lubinski D. Benbow C. (2009). Spatial ability for STEM domains: aligning over 50 years of cumulative psychological knowledge solidifies its importance. J. Educ. Psychol. 101, 817–835. doi: 10.1037/a0016127

[ref83] Yang J. Wu D. Luo J. Xie S. Chang C. Li H. (2020). Neural correlates of mental rotation in preschoolers with high and low working memory capacity: an fNIRS study. Front. Psychol. 11:568382. doi: 10.3389/fpsyg.2020.568382 33362634 PMC7758205

[ref84] Young C. J. Levine S. C. Mix K. S. (2018). The connection between spatial and mathematical ability across development. Front. Psychol. 9:755. doi: 10.3389/fpsyg.2018.00755, PMID: 29915547 PMC5994429

